# Dental Items

**Published:** 1871-01

**Authors:** 


### EDITORIAL DEPARTMENT.

*Dental Items.*—We have received from Dr. J. T. Love, through Dr. Jno. C. Storey, of Eutaw Ala., for the Museum of the Baltimore College of Dental Surgery, one of the finest specimens of osseous union of the teeth, which we have yet seen. It consists of two inferior incisors joined by complete union of both crowns and roots—from the cutting edges to the extremities of the roots. Also another valuable specimen of the same malformation from Dr. Wm. J. Fogle of Columbus, Ga., which consists of two molars, the superior second and third, joined by an osseous union of their roots. The roots of both teeth are enlarged—exostosed—by a deposit of osseous matter resembling enamel in color and density much more than cementum.

Dr. Jno. C. Storey writes that he now uses, at the suggestion of Dr. R. E. Watkins, of Eutaw, Ala. lead packing for vulcanizers, and prefers it to rubber packing.

Dr. Webster in the "Canada Journal of Dental Science" gives the following directions for drying cavities preparatory to introducing the material composing the filling: "In order to dry out a cavity, I use cotton wool and then dip a pledget of cotton in alcohol and wipe out the cavity with it, and it evaporates leaving the cavity as dry as spunk." "Chloroform has also been used for the same purpose."

Dr. Carroll in the "Medical Gazette" states: that "in September, 1868, Dr. Perrine, of this city, afforded me an opportunity of trying the spray apparatus upon a patient of his, who had, I think sixteen teeth and fangs, upper and lower, extracted without pain under its influence. (Vide *Medical Gazette*, September 19, 1868, p. 397). The use of the spray for three or four minutes sufficed to deaden sensation long enough for the extraction of all the teeth on the anæsthetized side, and probably long enough for the completion of the painful portion of excavating a sensitive tooth. In patients with much adipose tissue over the ganglion, the effect is less rapid and less profound; but even where sensation is not destroyed it is very much lessened. I write this to give a hint which I should be glad to learn has led to satisfactory results among your sensitive patients.

In the same journal the following directions are given for preserving instruments from rust: If polished instruments are rubbed with pulverized casistic lime, or dipped in lime milk, they do not rust. The lime is easily removed when the instrument is required for use."

Dr. A. Berry, in the "Dental Register" recommends "cones of pumice stone" for polishing rubber plates, the cone being kept wet and used immediately after the steel bur or scraper, and followed by pulverized pumice, or cork or leather cones, and lastly by a brush and whiting. The cone of pumice is made by sawing from the firm stone, in the direction of the grain, pieces an inch or more in diameter and two or more long. A tapering hole is made in one end, into which is fitted by liquid silex or other cements, a piece of wood by which the cone is attached to the lathe. After cutting off the angles the piece of pumice-stone is shaped into the form of a cone by holding firmly against it a large piece of the same stone and revolving rapidly.

